# Distribution of Pupil Size and Associated Factors: Results from the Population-Based Gutenberg Health Study

**DOI:** 10.1155/2022/9520512

**Published:** 2022-09-09

**Authors:** Marian Kiel, Stephanie D. Grabitz, Susanne Hopf, Thomas Koeck, Philipp S. Wild, Irene Schmidtmann, Karl J. Lackner, Thomas Münzel, Manfred E. Beutel, Norbert Pfeiffer, Alexander K. Schuster

**Affiliations:** ^1^Department of Ophthalmology, University Medical Center of the Johannes Gutenberg University Mainz, Mainz, Germany; ^2^Preventive Cardiology and Preventive Medicine, Department of Cardiology, University Medical Center of the Johannes Gutenberg University Mainz, Mainz, Germany; ^3^German Center for Cardiovascular Research (DZHK), Partner Site Rhine-Main, Mainz, Germany; ^4^Center for Thrombosis and Hemostasis (CTH), University Medical Center of the Johannes Gutenberg University Mainz, Mainz, Germany; ^5^Institute for Medical Biostatistics Epidemiology and Informatics (IMBEI), University Medical Center of the Johannes Gutenberg University Mainz, Mainz, Germany; ^6^Institute for Clinical Chemistry and Laboratory Medicine, University Medicine of the Johannes Gutenberg University Mainz, Mainz, Germany; ^7^Department of Cardiology, University Medicine of the Johannes Gutenberg University Mainz, Mainz, Germany; ^8^Department for Psychosomatic Medicine und Psychotherapy, University Medical Center of the Johannes Gutenberg University Mainz, Mainz, Germany

## Abstract

**Results:**

18,335 eyes of 9,559 participants aged 40 to 80 years were included in the analysis. Median pupil diameter was 4.19 mm in right eyes and 4.12 mm in left eyes. A smaller pupil was associated with older age, hyperopic refractive error, previous cataract surgery, diabetes, obesity, and ACE inhibitor intake, whereas wider pupil was associated with female gender, arterial hypertension, intake of tricyclic antidepressants, and intake of SNRI and tetracyclic antidepressants. Socioeconomic status and smoking were not associated with pupil size.

**Conclusion:**

Individuals of older age, after cataract surgery, under therapy with ACE inhibitors and with diabetes have a smaller pupil. This should be taken into account when planning nonmydriatic fundus photography-based screening programs, for instance, for diabetic retinopathy.

## 1. Introduction

The pupil regulates the incoming light to reduce glare, to control retinal illumination, and to achieve sufficient depth of field. Pupil size and its reaction are modulated by the parasympathetic and sympathetic parts of the autonomous nervous system [1].

Sufficient pupil width is essential in ophthalmologic diagnostic to assess the periphery of the retina and to achieve adequate imaging quality in screening examinations, such as diabetic retinopathy screening. The latter is performed in some countries using fundus photography and subsequent grading of the recorded images [2]. To record images with sufficient signal-to-noise ratio including illumination, contrast, and resolution, a minimum width of pupil size is necessary, even in nonmydriatic fundus photography. New approaches using AI algorithms evaluate these fundus images with regard to the presence and stage of diabetic retinopathy [3, 4]. Similarly, programs to detect glaucoma are under development [5]. Nevertheless, photographs with insufficient quality and poor focus are being excluded. To assess the large-scale feasibility of these programs, it is of interest whether special population groups would face disadvantages in miotic fundus photography due to their expected pupil size.

In the past, several conditions influencing mesopic pupil size were investigated. Diabetic adults have a significantly smaller pupillary diameter due to neuropathy of the sympathetic innervation of the musculus dilator pupillae as reported by Smith and Smith. According to the authors, this explains why pharmacological agents, which paralyze the parasympathetic musculus constrictor pupillae, are commonly known to be less effective in patients with diabetic neuropathy [6]. As the generally higher age of diabetes patients is said to contribute to miosis, the extent of miosis caused by diabetes is partially still under debate [7].

Furthermore, it is unclear whether refractive error is associated with mesopic pupil size. Guillon et al. stated that larger pupils occur in younger patients and myopes [8]. By contrast, Orr et al. reported pupil size to be determined by the pupillary light reflex but not by refraction [9]. The relation between pupil size and refractive error might be overestimated for two reasons according to Loewenstein and coauthors: firstly, refractive errors cause misperception of the pupil size, and secondly, the prevalence of myopia, emmetropia, and hyperopia differs depending on age groups [7].

In the past, researchers compared pupil size before and after ophthalmological surgery. Hayashi and Hayashi found that pupil size did not differ significantly before and after phacoemulsification [10]. By contrast, a change toward a smaller pupil and more circular appearance after phacoemulsification was reported by Kanellopoulos and Asimellis in 2014. They suspected that a change in pupil size correlates with increase of anterior chamber depth after surgery [11].

With regard to medication use, the nonselective beta-blocker carvedilol is known to cause miosis [12]; however, this has not yet been demonstrated with other beta-blockers. Tricyclic antidepressants are known to induce pupil dilation [13]. We are not aware of any population-based studies examining the effect of calcium channel inhibitors, thiazide diuretics, and ACE inhibitors on pupil size.

To our knowledge, no population-based study so far reported the distribution of pupil size and evaluated potential associations with systemic diseases, the peroral intake of drugs, and ocular characteristics. There are still some uncertainties in the literature on various variables influencing pupil size. Therefore, the aim of this investigation was to describe the distribution of pupil size and to examine associations with pupil size and diabetes, refractive error, and preceding ophthalmic surgery in the Gutenberg Health Study.

## 2. Materials and Methods

### 2.1. Study Population

The Gutenberg Health Study (GHS) is an observational, prospective, interdisciplinary, single-center, and population-based cohort study at the University Medical Center of the Johannes Gutenberg-University Mainz in Germany, enrolling 15,010 participants at aged 35 to 74 years at baseline examination between 2007 and 2012 [14]. Main goals of the ophthalmological section are to assess the prevalence and incidence of ocular diseases and associations with systemic diseases, as well as to provide the population-based distribution of ocular characteristics. A total of 12,423 participants were studied at the 5-year follow-up visit between 2012 and 2017, with a valid pupil size measurement in 9559 participants (76.9%). The presence of iris trauma, pseudoexfoliation syndrome, and iris claw intraocular lens implant were exclusion criteria.

Written informed consent was obtained from all study participants prior to their entry into the study. The GHS complies with Good Clinical Practice (GCP), Good Epidemiological Practice (GEP), and the ethical principles of the Declaration of Helsinki. The study protocol and study documents were approved by the local ethics committee of the Medical Chamber of Rhineland-Palatinate, Germany (reference no. 837.020.07; original vote: 22 March 2007, latest update: 20 October 2015).

### 2.2. Ophthalmologic Examination

During the ophthalmologic examination, pupil diameter was measured by noncontact optical biometry using the Lenstar LS900 (Haag-Streit, Koeniz, Switzerland) under mesopic light conditions. This device performs pupil measurement with the help of an infrared camera. According to Freedman et al., the size of the physical pupil is crucial for light refraction and for the image projected on the retina, not the clinically measured size. Therefore, the physical size of the pupil is also the relevant parameter for nonmydriatic fundus photography, while the description for nonmydriatic cameras refers to the clinical pupil [15, 16]. The physical pupil size was subsequently calculated to correct for optical phenomena due to anterior segment aberrations [15]

(1) $$\begin{array}{c} \text{PP}=\text{EP}\left(\frac{1-AK}{n_{2}}\right), \end{array}$$

where PP is physical pupil, EP is entrance pupil or clinically measured pupil, *A* is central anterior chamber depth, *K* is central corneal refractive power, and *n*_2_ is refractive index of the cornea (1.3375).

In addition, axial length, central corneal thickness, corneal curvature, white-to-white distance, anterior chamber depth, and lens thickness were determined. In previous studies, the results of the Lenstar LS 900 have been shown to be very accurately reproducible by the Visante AS-OCT and IOL-Master (Carl Zeiss AG, Jena, Germany) [17]. Scheimpflug imaging of the anterior segment was conducted with the Pentacam HR (Oculus, Wetzlar, Germany). Objective refraction was measured using a Humphrey Automated Refractor/Keratometer (HARK) 599 (Carl Zeiss AG, Jena, Germany). Distant-corrected visual acuity was recorded using the built-in Snellen charts. The spherical equivalent was calculated as the spherical correction value plus half the cylindrical power. Phakia and pseudophakia were determined based on Scheimpflug imaging as described before [18]. In a subsample, we determined the lens position of pseudophakia with respect to the pupil plane and incorporated this into the above-named formula. Intraocular pressure was measured with an air-puff noncontact tonometer (Nidek NT-2000; Nidek Co., Gamagori, Japan) with the mean value of three measurements within a 3-mmHg range being recorded [19]. Every participant was asked if they have had previous ocular surgery or suffer from any kind of glaucoma, macular degeneration, or other eye diseases.

General health conditions were surveyed in a computer-assisted personal interview. Smoking was dichotomized into nonsmokers (never smokers and ex-smokers) and smokers (occasional smokers and smokers). Arterial hypertension was diagnosed if antihypertensive drugs were taken or if the mean blood pressure was elevated above 140 mmHg (systolic) or 90 mmHg (diastolic) at the second and third standardized measurement after resting [20]. Diabetes was stated upon a respective diagnosis and treatment by a physician or if individuals showed HbA1c level ≥6.5%. Anthropometric measurements were performed with calibrated digital scales (Seca 862, Seca, Hamburg, Germany) and a measuring stick (Seca 220, Seca, Hamburg, Germany), and body mass index was computed. Obesity was diagnosed upon a BMI ≥30. A detailed description of the medical examinations in the Gutenberg Health Study has been published by Höhn et al. [19].

In addition, the socioeconomic status (SES) based on income, education, and occupation was surveyed. The SES index used ranges from 3 to 21 (3 indicates the lowest and 21 the highest SES) [21].

### 2.3. Statistical Analysis

Descriptive statistics were calculated for all primary and secondary variables. For categorical data, absolute and relative frequencies were computed. For continuous parameters, mean and standard deviation was calculated for all approximately normal distributed variables, otherwise median and interquartile range. Normative diagrams for the relation between pupil size and age were computed. Univariate and multiple linear regression analyses with generalized estimating equations were performed to evaluate associated factors with pupil size; using this approach, we adjusted the models for the inclusion of right and left eyes. The main model considered the participants' demographics and systemic diseases. This included age, gender, spherical equivalent (SEQ), pseudophakia, socioeconomic status, diabetes, obesity, arterial hypertension, and smoking. The influence of thiazide diuretics (ATC-code C03A), beta-blockers (ATC-code C07), calcium antagonists (ATC-code C08), ACE inhibitors (ATC-codes C09A and C09B), tricyclic antidepressants, and other antidepressant drugs (ATC-code N06AX) on pupil size was calculated using an association analysis adjusted for age, sex, and spherical equivalent. To further assess the association of ocular biometry on pupil size, we created another regression model in phakic eyes only considering central corneal thickness, corneal power, axial length, anterior chamber depth, lens thickness, white-to-white distance as surrogate parameter for corneal diameter, age, and gender as independent variables. Finally, we studied aspects influencing pupil size in a subcohort of participants with diabetes (age, gender, pseudophakia, HbA1c, duration of diabetes, and diabetic peripheral neuropathy). A sensitivity analysis was conducted excluding eyes with pseudoexfoliation as observed in slit-lamp examination at baseline examination. Quantile regression analysis (percentiles: 0.05; 0.25; 0.5; 0.75; 0.95) was used to further explore the association between age and pupil size. The data were analyzed with R version 3.6.1 [22].

## 3. Results

**Figure 1:**
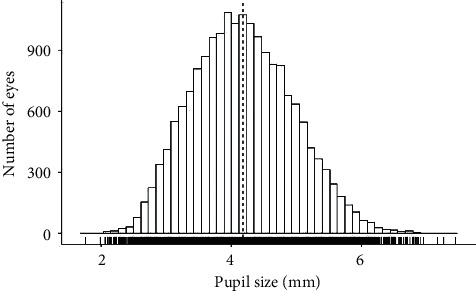
Distribution of mesopic pupil size in the general population in Germany. Data from the population-based Gutenberg Health Study in 2012–17 (*n* = 18,335).

**Figure 2:**
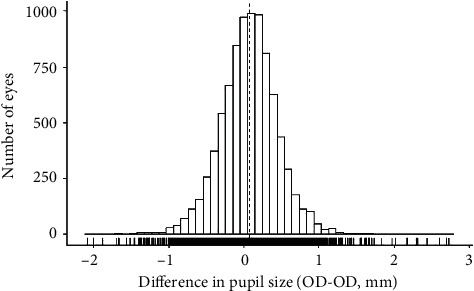
Difference in mesopic pupil sizes between both eyes. Data from the population-based Gutenberg Health Study in 2012–17 (*n* = 8,776).

18,335 eyes of 9,559 subjects aged 40 to 80 years were included in this study. The median age was 59 years, and the proportion of women was 49.0%. 7.3% of subjects were obese (BMI of ≥30). Arterial hypertension was present in 52.5% of individuals, and 9.6% had some form of diabetes. 15.3% were identified as smokers. Median mesopic pupil size was 4.19 mm in right eyes (Figure 1) and 4.12 mm in left eyes. Median spherical equivalent in both eyes was -0.12 diopters (Supplementary Table 1). The respective deviation of pupil sizes between the right and the left eye (anisocoria) is shown in Figure 2.

### 3.1. Main Regression Model

**Figure 3:**
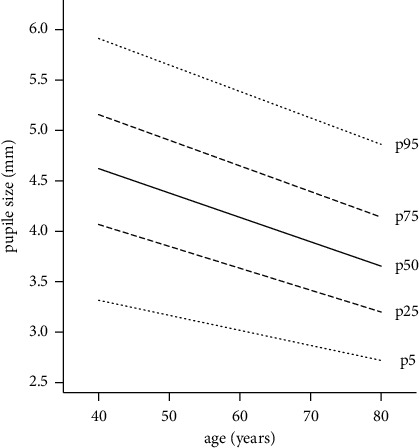
Influence of age on mesopic pupil size. Predicted mesopic pupil size is derived from quantile regression models. Data from the population-based Gutenberg Health Study in 2012–17 (*n* = 18,335 eyes).

In univariate analysis, an association of smaller pupil size and higher age, diabetes, BMI ≥30, arterial hypertension, higher spherical equivalent, and pseudophakia was found. For each 10 years of age, the pupil size becomes smaller by 0.2 mm (Figure 3). A larger pupil size was associated with female gender, higher socioeconomic status, and smoking.

In the first multivariable analysis, the association of smaller pupil size and higher age, diabetes, BMI ≥30, higher spherical equivalent, and pseudophakia remained robust (Table 1). In the second multivariable analysis comparing the systemic medication with sex, age, and spherical equivalent, we found that subjects taking ACE inhibitors had a significantly smaller pupil size (Table 2). A larger pupil size was associated with the intake of tricyclic antidepressants and other antidepressant drugs (SNRI and tetracyclic antidepressants). No significant effect on pupil size was demonstrated for calcium channel inhibitors and thiazide diuretics. A total of 35 participants showed signs of pseudoexfoliation. After excluding these participants, our primary analysis remained robust.

### 3.2. Ocular Geometry Model

In univariate analysis, a smaller pupil size was associated with higher age, male gender, higher corneal power, larger central corneal thickness, and larger lens thickness, respectively. A larger pupil size was associated with female gender, larger anterior chamber depth, larger white-to-white distance, and longer axial length.

In the multivariable analysis, the association of smaller pupil size and higher age, higher corneal power, and larger central corneal thickness remained robust. Furthermore, female gender, larger white-to-white distance, longer axial length, and higher intraocular pressure were independent predictors of larger pupil size (Table 3). We did not find an association between pupil size and lens thickness as well as between pupil size and anterior chamber depth after multivariable adjustment.

### 3.3. Diabetes Model

In the univariate analysis, smaller pupil size was associated with higher age, diabetes duration, and pseudophakia. There was no association between pupil size and HbA1c level and existing peripheral diabetic neuropathy (Supplementary Table 2).

In the multivariable analysis, we found a statistically significant association between smaller pupil size and higher age and pseudophakia. Female sex was associated with a larger pupil size. There was no association of pupil size and HbA1c level, diabetes duration, and existing peripheral diabetic neuropathy.

## 4. Discussion

Using our large epidemiological study, we were able to analyze the distribution of pupil size on population-based level. Persons of older age, after cataract surgery, with hyperopia and with diabetes have a smaller pupil on population-based level, which should be taken into account when setting up fundus photography-based screenings.

In our study, eyes with a hyperopic refractive error showed a smaller pupil size. Our data considered optical distortion due to corneal refractive power and anterior chamber depth [15] and thus was not influenced by optical magnification. A longer axial length was similarly associated with a larger pupil, as described by Cakmak et al. [23], Guillon et al. [8], and Hirsch and Weymouth [24]. The causes for these refraction-related differences in pupil size have been attributed at least in part to accommodation in hyperopic subjects when looking far, resulting in greater pupil contraction than their myopic counterparts.

In our study, a larger pupil diameter was related to a longer axial length, myopic refractive error, and larger white-to-white distance. Cakmak et al. stated that a general increase in the dimensions of an eye also implies a larger pupil diameter [25]. Contrarily, Loewenfeld and Lowenstein concluded from their studies that the relation between pupil size and refractive error is overestimated due to misperception of pupil size caused by the refractive errors, and due to myopia, emmetropia and hyperopia being prevalent in different age groups [7]. Our findings disagree with this view, as the dependence between pupil size and refractive error was shown in a multivariable linear regression analysis adjusting for age, sex, and systemic diseases.

Pupil size is smaller in older individuals [26], similar found in our study. As for the reasons, an age-related iris stroma stiffness and age-related loss of sympathetically caused inhibition of parasympathetic neuronal discharges in the midbrain and spinal cord seem to be essential [7]. Okamura and Lütjen-Drecoll noted an increase of collagen fibers of the iris when comparing iridectomy samples from young and old patients under an electron microscope [27], which may lead to increased stiffness of the iris stroma. Loewenfeld and Lowenstein illustrated the age-related loss of sympathetically caused inhibition of parasympathetic neuronal discharges with old people showing “fatigue signs” that can also be seen in young people, especially if they are tired. Accordingly, well-rested and calm young people show the lowest central inhibition among all age groups, resulting in a larger pupil diameter [7].

In our data, previous cataract surgery was linked to a smaller pupil size. Kanellopoulos and Asimellis explained that the ageing and more voluminous natural lens has a steeper slope on its surface that causes dilation and enlargement of the pupil. The thinner artificial intraocular lens causes the lens diaphragm to be displaced less anteriorly than the natural lens. Consequently, the iris flattens and the pupil becomes less dilated [11, 28]. Hayashi and Hayashi found pupil size before and after phacoemulsification not to be significantly different, stating that pupil size “returned to approximately preoperative levels by 1 month postoperatively” [10]. Based on the results of our population-based cohort study, we found a pupil size difference of 0.21 mm due to cataract surgery.

Loewenfeld mentioned that age partially contributes to the miosis in diabetic patients, as this specific cohort tends to be older than average [7]. We conducted an analysis adjusting for the aging effect and detected a smaller pupil size in diabetic subjects. Further analyses of pupil size within participants with diabetes showed an association between peripheral diabetic neuropathy and a smaller pupil size in the univariable but not in the multivariable model. Different than expected, there was no association between HbA1c level and duration of disease in the multivariable model.

It is reported that a measurement of pupil diameter shortly after tobacco smoking shows decreased values [29]. This is related to the stimulation of nicotinic acetylcholine receptors resulting in pupil constriction [29]. Nevertheless, our data do not support this relationship insofar as no association between smoking and decreased pupil size was evident in our large cohort.

Most studies have found no gender difference in pupil size [9, 30, 31]. By contrast, the presented population-based study showed (small) differences in male and female pupil size. However, the results are at odds with previous publications and need to be clarified by future studies.

Historically, there have been opposing views on the effect of arterial hypertension on pupil size. In 1915, Wiener formulated the opinion that has prevailed since then that arterial hypertension would cause a slight pupil enlargement [32]. A relationship between transient increases in blood pressure during breathing and transient increases in pupil size being transmitted through afferent carotid baroreceptor pathways as suggested by Calcagnini et al. might also be conceivable [33]. In our multivariable analysis, we reached the same conclusion.

We did not find a statistically significant relationship between pupil size and the intake of beta-blockers. This is of interest, as most patients in the GHS are known to take selective beta-blockers [34]; the observation of Hysek and Liechti of carvedilol being associated with smaller pupil size dealt with a nonselective beta-blocker [12]. It is known that 10% *β*1 receptors and 90% *β*2 receptors are present on the iris-ciliary body [35]. The cardioselective beta-blockers, which primarily target the *β*1-receptors, therefore have a reduced effect.

A new observation is that individuals taking ACE inhibitors have a smaller pupil size. In the few reports available, no influence of ACE inhibitors on pupil size was detected [36].

The fact that people taking tricyclic antidepressants have larger pupil size is consistent with our findings. Drugs under the ATC-code N06AX (SNRI and tetracyclic antidepressants) have been known to both cause pupil dilation, as in the cases of duloxetine and reboxetine [37, 38], and pupil constriction, as in the cases with mianserin [39, 40]. For future studies, a more precise subdivision of antidepressants into ATC codes would facilitate a population-based evaluation in terms of pupil size.

## 5. Strengths and Limitations

A major strength of our study is the very large cohort and its population-based sampling. In many past studies on the topic of pupil size and its influencing factors, the cohort size was below or slightly above 100 subjects. By including 9,559 individuals, even small differences in pupil size could be detected and associated factors determined.

Our study has several limitations. Firstly, our measurements were made under mesopic light conditions after an adaptation time of about 5 minutes. However, a nearly perfect adaptation can only be expected after 20 or more minutes [7]. We only measured pupil size under mesopic light conditions at one time and cannot make statements about pupil size at other light conditions. Furthermore, it was not determined how fast or slow the individual pupil of the respective subject reacted to changing light conditions. Another weak point is the age distribution of our cohort, since persons younger than 40 years and older than 80 years were not examined. In some subjects of the 5-year follow-up visit, the pupil size could not be measured due to technical difficulties. In all likelihood, this constitutes missing at random.

## 6. Conclusions

Persons of older age, after cataract surgery, with hyperopia, under antihypertensive drug treatment with ACE inhibitors and with diabetes have a smaller pupil. This should be considered when developing and assessing the feasibility of screening using nonmydriatic fundus photography as a sufficiently large pupil is required to achieve adequate image quality, especially when aiming to use AI algorithms for screening.

## Figures and Tables

**Table 1:** Association analysis of systemic parameters and pupil size. Data from the population-based Gutenberg Health Study in 2012–17 (*n* = 18, 176 eyes).

Parameters	Univariable			Multivariable		
	*B*	95% CI	*p*-value	*B*	95% CI	*p*-value
Age	−0.02	−0.02; −0.02	<0.001	−0.02	−0.02; −0.02	<0.001
Sex (female)	0.04	0.02; 0.06	<0.001	0.02	0.00; 0.05	0.01
SES	0.02	0.02; 0.02	<0.001	0.00	0.00; 0.00	0.86
Diabetes	−0.23	−0.26; −0.19	<0.001	−0.07	−0.10; −0.03	<0.001
Obesity	−0.06	−0.09; −0.04	<0.001	−0.03	−0.05; 0.00	0.01
Arterial hypertension	−0.19	−0.21; −0.16	<0.001	0.02	0.00; 0.05	0.05
Smoking	0.08	0.05; 0.11	<0.001	−0.02	−0.05; 0.01	0.2
Spherical equivalent	−0.04	−0.04; −0.03	<0.001	−0.02	−0.02; −0.01	<0.001
Pseudophakia	−0.45	−0.49; −0.40	<0.001	−0.16	−0.21; −0.12	<0.001

*B* = regression coefficient, CI = confidence interval, SES = socioeconomic status, *n* = number of subjects.

**Table 2:** Association analysis of antihypertensive drug intake and pupil size. Data from the population-based Gutenberg Health Study in 2012–17 (*n* = 16, 266 eyes).

Parameters	Multivariable		
	*B*	95% CI	*p*-value
Sex (female)	0.02	0.00; 0.04	0.1
Age	−0.02	−0.02; −0.02	<0.001
Spherical equivalent	−0.01	−0.02; −0.01	<0.001
Calcium antagonists	0.02	−0.02; 0.06	0.352
ACE-inhibitors	−0.04	−0.07; −0.02	0.001
Beta-blockers	−0.002	−0.03; 0.03	0.862
Thiazide diuretics	−0.03	−0.09; 0.01	0.138
SSNRI, SNRI, and tetracyclic antidepressants	0.33	0.25; 0.41	<0.001
SSRI	−0.007	−0.08; 0.06	0.847
Tricyclic antidepressants	0.11	0.05; 0.19	<0.001

*B* = regression coefficient, CI = confidence interval, *n* = number of subjects.

**Table 3:** Association analysis of ocular parameters and pupil size in phakic subjects. Data from the population-based Gutenberg Health Study in 2012–17 (*n* = 16, 869 eyes).

Parameters	Univariable			Multivariable		
	*B*	95% CI	*p*-value	*B*	95% CI	*p*-value
Age	−0.02	−0.02; −0.02	<0.001	−0.02	−0.02; −0.02	<0.001
Sex (female)	0.05	0.02; 0.07	<0.001	0.09	0.07; 0.12	<0.001
Intraocular pressure (mmHg)	0.00	0.00; 0.00	0.81	0.01	0.01; 0.02	<0.001
Central corneal thickness (10 *μ*m)	−0.01	−0.01; −0.01	<0.001	−0.02	−0.02; −0.01	<0.001
Corneal power (Diopters)	−0.06	−0.07; −0.06	<0.001	−0.03	−0.04; −0.02	<0.001
White-to-white distance (mm)	0.32	0.29; 0.34	<0.001	0.17	0.14; 0.20	<0.001
Anterior chamber depth (mm)	0.26	0.23; 0.30	<0.001	0.04	−0.01; 0.09	0.15
Lens thickness (mm)	−0.32	−0.36: −0.29	<0.001	0.03	−0.01; 0.07	0.16
Axial length (mm)	0.07	0.06; 0.08	<0.001	0.02	0.01; 0.03	0.005

*B* = regression coefficient, CI = confidence interval, SES = socioeconomic status, *n* = number of subjects.

## Data Availability

The authors confirm that the data supporting the findings of this study are available within the article, tables, and its supplementary materials.

## Supplementary Materials

Supplementary Materials Supplementary Table 1: study characteristics of the analysis sample. Data from the population-based Gutenberg Health Study in 2012–17. *n* = number of subjects, IQR = interquartile range, BMI = body mass index, OD = right eye, OS = left eye. Supplementary Table 2: association analysis within participants with diabetes. Data from the population-based Gutenberg Health Study in 2012–17 (*n* = 1,321 eyes). *B* = regression coefficient, CI = confidence interval, *n* = number of subjects.
