# Supplementary material for: Distribution of Pupil Size and Associated Factors: Results from the Population-Based Gutenberg Health Study
Source: J Ophthalmol. 2022 Sep 9;2022:9520512. doi: 10.1155/2022/9520512 (PMC9481399; doi:10.1155/2022/9520512)
Supplement: Supplementary Materials — Supplementary Table 1: study characteristics of the analysis sample. Data from the population-based Gutenberg Health Study in 2012–17. n = number of subjects, IQR = interquartile range, BMI = body mass index, OD = right eye, OS = left eye. Supplementary Table 2: association analysis within participants with diabetes. Data from the population-based Gutenberg Health Study in 2012–17 (n = 1,321 eyes). B = regression coefficient, CI = confidence interval, n = number of subjects. [file 9520512.f1.docx]

The supplementary tables 1 and 2 show firstly study characteristics of the analysis sample and, secondly, an association analysis within participants with diabetes. The data have been retrieved from the population-based Gutenberg Health Study in 2012-17 (n = 1,321 eyes).

| Characteristics | n = 9559 |
| --- | --- |
| Sex (female) | 4677 (49%) |
| Age at examination (years) [Median, IQR] | 59.00 [50.00, 68.00] |
| Socio-economic status [IQR] | 13.00 [10.00, 17.00] |
| BMI [Median, IQR] | 26.65 [24.03, 30.05] |
| BMI ≥ 30 | 694 (7.3%) |
| Arterial hypertension | 5016 (52.5%) |
| Diabetes total | 919 (9.6%) |
| - Type 1 diabetes | 39 (0.4%) |
| - Type 2 diabetes | 676 (7.1%) |
| - Unspecified diabetes | 204 (2.1%) |
| HbA1c [Median, IQR] | 5.60 [5.30, 5.80] |
| Smokers | 1463 (15.3%) |
| Spherical equivalent OD (diopters) [Median, IQR] | -0.12 [-1.25, 0.88] |
| Spherical equivalent OS (diopters) [Median, IQR] | -0.12 [-1.25, 0.88] |
| Axial length OD (mm) [Median, IQR] | 23.62 [22.96, 24.39] |
| Axial length OS (mm) [Median, IQR] | 23.62 [22.96, 24.39] |
| White-to-white distance OD (mm) [Median, IQR] | 12.20 [11.92, 12.49] |
| White-to-white distance OS (mm) [Median, IQR] | 12.21 [11.92, 12.49] |
| Corneal power OD (diopters) [Median, IQR] | 43.52 [42.53, 44.50] |
| Corneal power OS (diopters) [Median, IQR] | 43.58 [42.56, 44.55] |
| Anterior chamber depth OD (mm) [Median, IQR] | 3.27 [3.02, 3.54] |
| Anterior chamber depth OS (mm) [Median, IQR] | 3.26 [3.02, 3.52] |

*Supplementary Table 1: Study characteristics of the analysis sample. Data from the population-based Gutenberg Health Study in 2012-17. n = number of subjects, IQR = interquartile range, BMI = body mass index, OD = right eye, OS = left eye.*

|  | Univariable | | | Multivariable | | |
| --- | --- | --- | --- | --- | --- | --- |
| Parameters | B | 95%-CI | p-value | B | 95%-CI | p-value |
| Age | -0.02 | -0.02; -0.02 | < 0.001 | -0.02 | -0.02; -0.01 | < 0.001 |
| Sex (female) | 0.07 | -0.01; 0.15 | 0.11 | 0.08 | 0.00; 0.16 | 0.042 |
| HbA1c | -0.03 | -0.06; 0.01 | 0.20 | -0.03 | -0.07; 0.01 | 0.14 |
| Duration of diabetes (years) | -0.01 | -0.01; 0.00 | < 0.001 | -0.002 | -0.01; 0.00 | 0.50 |
| Peripheral diabetic neuropathy | -0.11 | -0.22; 0.00 | 0.05 | -0.05 | -0.16; 0.05 | 0.33 |
| Pseudophakia | -0.29 | -0.40; -0.17 | < 0.001 | -0.14 | -0.26; -0.03 | 0.012 |

*Supplementary Table 2: Association analysis within participants with diabetes. Data from the population-based Gutenberg Health Study in 2012-17 (n = 1,321 eyes). B = regression coefficient, CI = confidence interval, n = number of subjects.*
